# Study on the Constitutive Model of Desert Sand–Fly Ash Cenosphere Lightweight Aggregate Concrete Under Steel Tube Confinement

**DOI:** 10.3390/ma19194068

**Published:** 2026-09-23

**Authors:** Changyue Luan, Junlin Guo, Ying Wang, Kang Yuan, Mingsheng He, Jianjiang Xu

**Affiliations:** 1 College of Water Conservancy and Architectural Engineering, Shihezi University, Shihezi 832003, China; 2 Xinjiang Production & Construction Corps Key Laboratory of Civil Engineering Structural Safety in Cold, Arid and Strong Earthquake Regions, Shihezi 832003, China

**Keywords:** desert sand–fly ash cenosphere lightweight aggregate concrete (DFCC), confined DFCC, constitutive model, DFCC-filled steel tube column, axial compression behavior, finite element analysis

## Abstract

**Highlights:**

- Hu model fitted unconfined DFCC curves well, with R^2^ = 0.9646~0.9794.
- Steel tube confinement improved DFCC strength and post-peak deformation.
- The modified model predicted stub-column capacity with a mean test-to-FE ratio of 1.02.
- The model enables nonlinear analysis of DFCC under steel tube confinement.
- DFCC combines lightweight performance with desert sand and cenosphere utilization.
- The study supports the use of DFCC in lightweight steel tubular composite members.

**Abstract:**

The compressive behavior of desert sand–fly ash cenosphere lightweight aggregate concrete (DFCC) under steel tube confinement differs from that of conventional lightweight aggregate concrete, and existing constitutive models may not adequately characterize its stress–strain response. This study aims to develop a compressive constitutive model for DFCC under steel tube confinement and evaluate its applicability to the axial response analysis of DFCC-filled steel tube stub columns. Uniaxial compression tests were first conducted to characterize the complete stress–strain response of unconfined DFCC, followed by finite element analyses of DFCC under different levels of steel tube confinement. Axial compression tests and numerical simulations of five DFCC-filled steel tube stub columns were further used for component-level evaluation. The results showed that unconfined DFCC exhibited pronounced post-peak softening, and the Hu model reproduced the measured complete stress–strain curves with coefficients of determination ranging from 0.9646 to 0.9794. Steel tube confinement enhanced the compressive resistance, deformation capacity, and post-peak residual strength of DFCC. Based on the confined stress–strain responses, a modified constitutive model was established by recalibrating the peak parameters and post-peak branch. When implemented in the finite element analysis of the stub columns, the ratios of experimental to numerical ultimate loads ranged from 0.97 to 1.06, with a mean value of 1.02 and a standard deviation of 0.035. The proposed model can therefore provide a practical constitutive description for the axial analysis of DFCC under steel tube confinement within the investigated parameter range.

## 1. Introduction

Driven by the national “dual-carbon” goals and the growing demand for green construction, the utilization of local resources and industrial by-products, together with the lightweight design of concrete, has received increasing attention in civil engineering [1]. The use of alternative resources and waste-derived materials in cementitious composites can reduce the consumption of conventional natural resources while providing an effective route for resource recycling [2]. Recent studies on various alternative and waste-derived constituents have further demonstrated the feasibility of this approach in the development of sustainable cement-based materials [3,4]. Northwest China is rich in desert sand resources, providing favorable conditions for its partial substitution for natural river sand [5,6,7]. Desert sand is much finer than conventional river sand, and excessive replacement may reduce concrete workability. Fly ash cenospheres, with a hollow spherical morphology and a substantially lower density than natural sand, can serve as a lightweight fine particulate component [8,9]. Xu et al. [10] investigated the combined replacement of river sand with desert sand (DS) and fly ash cenospheres (FAC) in ceramsite concrete. The slump decreased with increasing DS content but increased with increasing FAC content. The compressive strength first increased and then decreased as the DS replacement ratio increased, whereas it first decreased and then increased with increasing FAC content. Based on workability and mechanical performance, suitable replacement ratios of 20 vol% for DS and 30 vol% for FAC were identified. Microstructural observations also showed that the incorporation of DS and FAC promoted hydration product formation and improved the concrete microstructure. The combined use of DS and FAC therefore provides a means of balancing workability, mechanical performance, and lightweight requirements while reducing natural river-sand consumption. Desert sand–fly ash cenosphere lightweight aggregate concrete (DFCC) thus offers a potential route for the combined utilization of local desert resources and industrial by-products in lightweight structural applications [10,11].

Research on desert sand concrete, fly ash cenosphere concrete, and lightweight aggregate concrete has addressed a range of material properties. Yang et al. [12] examined the mechanical, durability, economic, and environmental performance of desert ultra-high-performance concrete, and Zhang et al. [13] investigated the mixture design of desert sand–ceramsite concrete. Zhang et al. [14] evaluated the rheological behavior, shrinkage, mechanical properties, and microstructure of cenosphere-based lightweight concrete. Guo et al. [15] further studied the shrinkage and creep behavior of lightweight aggregate concrete containing desert sand and fly ash cenospheres. These studies have provided a basis for understanding the mixture design, basic mechanical properties, microstructure, and long-term behavior of this type of concrete. By comparison, its complete compressive stress–strain response has received less attention [16,17]. In particular, the post-peak response and strength degradation remain insufficiently characterized, although both are important for describing the nonlinear compressive behavior of DFCC and establishing its constitutive relationship under lateral confinement.

Steel tube confinement can restrain the lateral expansion of core concrete and create a multiaxial compressive stress state, improving its compressive resistance and deformation capacity [18,19,20]. When lightweight aggregate concrete is used as the core material, the self-weight of concrete-filled steel tubular members can also be reduced while retaining the advantages of steel–concrete composite action [18,19,20]. For this reason, steel tube confinement was adopted in this study, with DFCC intended as the core material of steel tubular composite members. Zhang et al. [21] investigated steel tubular stub columns filled with stone-based lightweight aggregate concrete and examined the influence of lightweight aggregate replacement on axial compressive behavior. Xiao et al. [22] studied the eccentric compression behavior of self-compacting lightweight aggregate concrete-filled steel tubular columns, and Al-Eliwi et al. [23] investigated circular steel tubes filled with reinforced lightweight aggregate concrete under axial loading. Finite element methods have also been used to reproduce the nonlinear response of concrete-filled steel tubular members [24]. Since the confinement effect is ultimately reflected in the stress–strain response of the core concrete, an appropriate constitutive description is necessary for nonlinear analysis of steel tubular composite members [25].

A number of constitutive models have been proposed for confined concrete. Mander et al. [26] developed a classical stress–strain model for concrete confined by transverse reinforcement, providing a basis for describing the enhancement of concrete strength and deformation capacity under confinement. Han et al. [27] investigated the monotonic and cyclic behavior of steel-tube-confined concrete and demonstrated the influence of steel tube confinement on the axial response of core concrete. Yang [28] proposed a piecewise compressive constitutive relationship for lightweight aggregate concrete. Li et al. [29] later conducted monotonic axial compression tests on lightweight aggregate concrete confined by circular steel tubes with different diameter-to-thickness ratios and steel grades, and proposed expressions for the confined compressive stress, strain, and complete stress–strain relationship. These models provide an important basis for describing conventional and lightweight aggregate concrete under confinement.

Based on the literature review, constitutive models for conventional concrete and lightweight aggregate concrete under compression have been relatively well established. It is also recognized that the compressive stress–strain response is strongly influenced by aggregate properties. However, no study has yet proposed a compressive constitutive model for DFCC under steel tube confinement, and the applicability of existing models to DFCC remains unknown. To address this gap, the present study focuses on developing a compressive constitutive model for confined DFCC. Uniaxial compression tests were first conducted on DFCC specimens to obtain the complete stress–strain curves, and an existing uniaxial model was selected and modified based on the test data. This modified uniaxial model was then incorporated into finite element analyses of DFCC under different confinement levels, and the stress–strain relationships of confined DFCC were extracted. Based on these numerical results, a compressive constitutive model specifically applicable to confined DFCC was established. Finally, the proposed model was validated through axial compression tests on DFCC-filled steel tube stub columns. The findings of this study are expected to facilitate the application of this lightweight material in engineering practice.

## 2. Constitutive Model of DFCC Under Uniaxial Compression

### 2.1. Materials

Three groups of cylindrical concrete specimens with different mixture compositions were prepared, each with dimensions of 150 mm × 300 mm. Shale ceramsite with a particle size of 5–20 mm was used as the coarse aggregate. The fine aggregates consisted of river sand, desert sand, and fly ash cenospheres, which were obtained from the Manas River in Xinjiang, the southern margin of the Gurbantunggut Desert, and Henan Borun Foundry Materials Co., Ltd. (Zhengzhou, China), respectively. Their main physical properties are listed in Table 1. P·O 42.5 ordinary Portland cement (Xinjiang Tianye Cement Co., Ltd., Shihezi, China) and Class II fly ash (Xinjiang Yuelongda Renewable Resources Technology Co., Ltd., Shihezi, China) were used as cementitious materials, and a polycarboxylate-based high-range water-reducing admixture (Shihezi Tianchang Building Materials Co., Ltd., Shihezi, China) was used to adjust the workability of the mixtures. The polymer emulsion had a solid content of 40% and was incorporated to improve the interfacial bonding between the lightweight aggregate and the cementitious matrix. The water-reducing admixture had a nominal water-reduction rate of 15% and was used to maintain adequate workability at a relatively low water-to-binder ratio.

The mix proportions of the three concrete groups are summarized in Table 2. Ref denotes the reference group, which does not contain desert sand, fly ash cenospheres, or polymer emulsion. D30F30-LC25 and D20F30-LC30 represent two categories of lightweight aggregate concrete specimens. Specifically, D30 and D20 refer to desert sand replacement ratios of 30% and 20%, respectively; F30 signifies a fly ash cenosphere replacement ratio of 30%; and LC25 and LC30 indicate the corresponding strength grades.

### 2.2. Loading and Measurement Scheme

Three cylindrical specimens with dimensions of 150 mm × 300 mm were fabricated for each mix proportion for uniaxial compression tests in accordance with the Chinese Standard for Test Methods of Concrete Physical and Mechanical Properties (GB/T 50081–2019) [30]. For the material-level tests, a 5000 kN electro-hydraulic servo testing machine (Tianshui Hongshan Testing Machine Co., Ltd., Tianshui, China) applied load to the 150 mm × 300 mm cylindrical specimens. The cylinder loads were comparatively low. An independent 500 kN force sensor (Anhui Tianguang Sensor Co., Ltd., Hefei, China) therefore supplemented the built-in load-measurement system to improve the accuracy of axial load measurement over this lower range. Axial deformation was monitored using two symmetrically arranged LVDTs (Liyang Jincheng Testing Instrument Factory, Liyang, China) with a measurement range of 100 mm. The LVDTs were installed on the deformation-measuring frame with an axial gauge length L_0_ of 150 mm, in accordance with GB/T 50081–2019. The axial deformation used for strain calculation was taken as the average value measured by the two LVDTs, as shown in Figure 1. A rigid steel bearing plate was placed between the specimen and the force sensor. Prior to the formal loading phase, the specimen was preloaded to 0.2 times the cubic compressive strength (*f*_cu_) to eliminate interfacial gaps and ensure uniform loading. Following this, displacement-controlled loading was performed at a rate of 0.2 mm/min. This loading rate was selected to allow stable measurement of the complete stress–strain response, particularly the post-peak descending branch.

### 2.3. Test Results

Figure 2 shows the failure modes of the three groups of DFCC specimens under uniaxial compression. All three mixtures exhibited longitudinal and inclined cracks typical of compression failure. Compared with Ref-LC25, surface cracking was less pronounced in both D30F30-LC25 and D20F30-LC30, and the specimens retained better integrity after failure. According to Xu et al. [10], an appropriate amount of fine desert sand can improve particle packing, while fly ash cenospheres exhibit relatively good interfacial bonding with the surrounding mortar and promote the formation of hydration products near the interface. These features may contribute to a more uniform matrix and reduce the localization of compressive damage.

Figure 3 shows representative complete stress–strain curves of the three concrete groups under uniaxial compression. For each mixture, a curve containing both the ascending and post-peak branches was selected to represent its overall compressive response. The peak stresses of Ref-LC25, D30F30-LC25, and D20F30-LC30 were 22.73, 21.12, and 24.41 MPa, respectively, and the corresponding peak strains were 0.00202, 0.00234, and 0.00246. Their elastic moduli were 30.04, 29.70, and 31.11 GPa. The peak stress of D20F30-LC30 was 15.6% higher than that of D30F30-LC25. Xu et al. [10] showed that the workability and mechanical properties of DFCC depend on the relative proportions of desert sand, fly ash cenospheres, and polymer emulsion, and identified 20 vol% desert sand and 30 vol% fly ash cenospheres as a favorable combination within the investigated range. The higher peak stress of D20F30-LC30 may be related to the combined effects of its mixture design. This may be because the balance among the fine aggregates and cementitious paste promotes more suitable particle packing, while the micro-filling effect of fine desert sand and the distribution of hydration products contribute to a relatively denser matrix.

(1) $$\sigma =\frac{P}{A}$$

(2) $$\varepsilon =\frac{\Delta L}{L_{0}}$$

where *σ* is the axial compressive stress, *P* is the axial load acting on the specimen, *A* is the initial cross-sectional area of the specimen, *ε* is the axial strain, Δ*L* is the axial deformation of the specimen, and *L*_0_ is the gauge length of the displacement transducer.

### 2.4. Uniaxial Compressive Constitutive Model

Stress–strain curves predicted by five existing models were compared with the experimental results to establish a uniaxial compressive constitutive model suitable for DFCC. The key parameters of these models are summarized in Table 3.

In Table 3, *x* and *y* denote the normalized strain and normalized stress, respectively. For the piecewise models, *y*_1_ and *y*_2_ denote the normalized stress functions for the ascending and descending branches of the stress–strain curve, respectively.

A comparison between the experimental stress–strain curves and the predictions of various constitutive models is presented in Figure 4. The Carreira-J model and the Guo model exhibit relatively large deviations in the ascending branch, while the Hu model and the Dong model demonstrate better alignment in this region. In the descending branch, the Hu model shows the best fitting performance. The coefficients of determination (R^2^) for the full stress–strain curves predicted by the Hu model were 0.9646, 0.9734, and 0.9794 for Ref-LC25, D30F30-LC25, and D20F30-LC30, respectively. These results indicate that the Hu model can reasonably describe the stress–strain response of DFCC.

The Hu model given in Equation (3) was adopted as the basic constitutive framework for describing the uniaxial compressive behavior of DFCC. Based on the experimental stress–strain curves, regression analysis was conducted to determine the model parameters for each mixture, as listed in Table 4. The fitting results show that the R^2^ values for Ref-LC25, D30F30-LC25, and D20F30-LC30 are 0.9646, 0.9734, and 0.9794, respectively, with an average value of 0.9725. The consistently high R^2^ values indicate that the Hu model provides a reliable representation of the complete compressive stress–strain response of DFCC with different mixture proportions and strength grades.

(3) $$y=\frac{ax+bx^{2}}{1+cx+dx^{2}}$$

where *x = ε/ε*_0_; *y = σ*/*σ*_0_; *a*, *b*, *c*, and *d* are constants.

## 3. Constitutive Model of Confined DFCC

### 3.1. Stress–Strain Behavior of Confined DFCC

The unconfined relationship established in Section 2.4 describes DFCC without lateral restraint, whereas steel-tube confinement changes its peak stress, peak strain, and post-peak response. Finite element models were therefore developed to obtain the stress–strain curves of the core DFCC at different confinement levels, as shown in Figure 5.

Each model consisted of a 150 mm diameter, 300 mm high DFCC cylinder enclosed by a Q235 steel tube with a thickness of 3, 5, or 8 mm. The tube was represented by four-node reduced-integration shell elements (S4R), and the core DFCC by eight-node reduced-integration solid elements (C3D8R). A 15 mm element size was used throughout the model. Elzeadani et al. [36] used this element size in analyses that included circular specimens 150 mm in diameter with a length-to-diameter ratio of 2. Their mesh-sensitivity study showed that sizes up to 15 mm gave good predictions, whereas meshes coarser than 20 mm reduced the calculated axial capacity.

Surface-to-surface contact was defined between the steel tube and core DFCC. Hard contact was used in the normal direction and Coulomb friction in the tangential direction. The friction coefficient remained 0.6 in all nine models analyzed. This value is common in CFST simulations and agrees with the steel–concrete interface data discussed by Tao et al. [37]. The core DFCC was modeled using the Concrete Damaged Plasticity (CDP) model in ABAQUS 2021, and the compressive stress–strain relationship of DFCC in this model adopted the modified Hu model and corresponding parameters in Section 2.4. The dilation angle was 35° for Ref-LC25 and 30° for D30F30-LC25 and D20F30-LC30. The eccentricity, *f*_b0_/*f*_c0_, *K*_c_, and viscosity parameter were 0.1, 1.16, 0.6667, and 0.0001, respectively. No predefined triaxial stress–strain curve was assigned; the multiaxial response developed from the lateral expansion of the core DFCC and the passive confinement provided by the steel tube. The peak stresses were 22.73, 21.12, and 24.41 MPa for Ref-LC25, D30F30-LC25, and D20F30-LC30, respectively. The Hu model parameters are listed in Table 4. Each mixture therefore retained its own measured compressive response curve. The constitutive relationship for Q235 steel followed Ref. [38] throughout.

All translational degrees of freedom were restrained at the lower end, and axial displacement was applied to the upper surface of the core DFCC. The steel tube was not loaded axially at either end. Instead, it provided passive lateral restraint as the core expanded. This arrangement represents concrete under steel tube confinement rather than a conventional CFST member, in which the steel tube and concrete are loaded together.

The resulting confined DFCC curves are presented in Figure 6. Increasing the tube thickness from 3 to 5 and 8 mm raised the stress level and flattened the post-peak branch. Stress decreased relatively rapidly after the peak in the T3 models, whereas the T5 and T8 models retained higher stress at large strains. This trend reflects the stronger lateral restraint provided by the thicker steel tube.

### 3.2. Constitutive Modeling of Confined DFCC

The constitutive model proposed by Han et al. [27] provides a basis for describing the compressive response of steel-tube-confined concrete. Yang [28] subsequently developed a constitutive relationship for lightweight aggregate concrete under steel tube confinement. In the present study, the stress–strain responses obtained for confined DFCC were used to modify the existing formulation according to the material characteristics of DFCC. Finite element analysis was conducted to obtain the stress–strain curves of confined DFCC, which served as the fundamental data for parameter calibration. Instead of developing a brand-new constitutive function, this study compared the prediction results of the conventional confined lightweight aggregate concrete model with the numerical stress–strain curves. Key parameters that control the peak mechanical performance and post-peak behavior of confined concrete were adjusted to accommodate the unique mechanical properties of DFCC. The ascending branch was described using a rational fraction function, while the post-peak branch was defined according to the confinement coefficient. The corresponding equations are presented below.

Peak stress of confined DFCC:

(4) $$\sigma_{o}=f_{c}^{\prime }\left[1.21+{\left(\frac{13}{f_{c}^{\prime }}\right)}^{0.45}\left(-0.2742\theta^{2}+0.5789\theta \right)\right]$$

Equation (4) retains the functional form of the peak-stress relationship proposed by Yang [28]. The original coefficients 1.194, −0.07485, and 0.5789 were recalibrated as 1.21, −0.2742, and 0.5789 for DFCC.

(5) $$\theta =\frac{f_{y}A_{s}}{f_{c}^{\prime }A_{c}}$$

where *f*_y_ and *A*_s_ are the yield strength and cross-sectional area of the steel tube, and *f’*_c_ and *A*_c_ are the axial compressive strength and cross-sectional area of the core DFCC.

Peak strain of unconfined DFCC:

(6) $$\varepsilon_{cc}=\left(1793.36+17.91f_{c}^{\prime }\right)\times 10^{-6}$$

Peak strain of confined DFCC:

(7) $$\varepsilon_{o}=\varepsilon_{cc}+\left[1000+800\left(\frac{f_{c}^{\prime }-24}{24}\right)\right]\theta^{0.2}$$

The ascending branch (*x* ≤ 1) reflects the initial elastic stage of loading and the subsequent elastoplastic evolution:

(8) $$y=\frac{ax+bx^{2}}{1+cx+dx^{2}}$$

where *a* = *E*_c_*ε*_o_*/f’*_c_, *b* = [(*a* − 1)^0.5^/0.27] − 1, *c* = *a* − 2, *d* = 0.21 − *b, E*_c_ = 4700*√f’*_cu,k_

In the descending branch, the influence of the confinement coefficient on the post-peak response was examined. The characteristics of stress degradation, plateau, or hardening under varying levels of confinement were analyzed separately to illustrate the mechanical evolution of DFCC in the post-peak stage when confined by a steel tube. Following the piecewise constitutive framework proposed by Yang [28] for steel-tube-confined lightweight aggregate concrete, *θ* = 1.22 was retained as the transition value separating the two forms of post-peak response. Since DFCC is a type of lightweight aggregate concrete and the present model was developed through modification and recalibration of this existing framework, the same transition value was adopted in the present study.

When *θ* ≥ 1.22, the curve exhibits a stable plateau or subsequent strain hardening:

(9) $$y=1-q+qx^{0.1\theta }$$

where *q* is the strengthening coefficient related to the confinement effect and *k* is a coefficient associated with the confinement coefficient *θ*. It is defined as *q* = *k*/(0.2 + 0.1*θ*), where *k* = 0.1*θ*^0.745^

When *θ* < 1.22, the stress degradation process is described by the following equation:

(10) $$y=\frac{x}{\beta {\left(x-1\right)}^{2}+x}$$

where *β* is the shape parameter of the descending branch, reflecting the post-peak stress degradation rate. Its expression is given by:

(11) $$\beta ={\left(2.36\times 10^{-5}\right)}^{\left[0.25+{\left(\theta -0.5\right)}^{7}\right]}\times 5\times f_{c}^{\prime 2}\times 10^{-4}$$

## 4. Constitutive Model Validation of Confined DFCC

### 4.1. Evaluation of Model Fitting at the Material Level

Figure 7 compares the stress–strain curves calculated using the modified constitutive model with the confined DFCC curves obtained from the finite element analyses. The model closely follows the numerical responses in the ascending branch and near the peak, while also reproducing the changes in post-peak degradation and residual strength with confinement level. The same numerical curves were used to calibrate the model parameters; Figure 7 therefore shows how well the model fits the calibration data. Comparison with the independent DFCC-filled steel tube stub-column test results is presented in Section 4.2.

### 4.2. Component-Level Experimental Validation

To further validate the proposed constitutive model for confined DFCC at the component level, axial compressive tests and finite element analyses were conducted on five DFCC-filled steel tube stub columns. The design parameters of the specimens are detailed in Table 5. The parameters used in these models were consistent with those described in Section 3.2, while the loading method was modified to ensure that both the steel tube and the core concrete were subjected to simultaneous loading. The material properties of the core concrete were defined using the previously proposed compressive constitutive model of confined DFCC.

Within the D30F30 series, the increase in steel tube thickness was accompanied by an increase in both the confinement coefficient and the ultimate bearing capacity. When the tube thickness increased from 3 mm to 5 mm and 8 mm, the confinement coefficient θ increased from 0.93 to 1.18 and 1.43, while the ultimate bearing capacity increased from 2055.88 kN to 2589.31 kN and 3054.71 kN, respectively. Compared with D30F30-Q235-T3, the increases in θ were 26.9% and 53.8%, and the corresponding increases in ultimate capacity were 25.9% and 48.6%. This trend is consistent with the expected role of the thicker steel tube in providing stronger lateral restraint to the core DFCC.

The influence of the axial compressive strength of the core DFCC can be examined by comparing D30F30-Q235-T3 and D20F30-Q235-T3. The cylindrical axial compressive strength of the core DFCC increased from 21.12 MPa to 24.41 MPa, with an increase of 15.6%, while the ultimate bearing capacity increased from 2055.88 kN to 2605.32 kN, with an increase of 26.7%. The comparison indicates that the increase in the axial compressive strength of the core DFCC contributed to the improvement in the axial bearing capacity of the DFCC-filled steel tube stub column.

Figure 8 illustrates a comparison between the experimental failure modes and the finite element stress contour plots for all specimens. The finite element results align well with the experimental observations, effectively reflecting the failure characteristics of the specimens and the distribution pattern of high-stress regions. In thin-walled specimens, local deformation was primarily concentrated in the middle of the column, where significant outward bulging was observed during testing; correspondingly, stress concentration was noted at the same location in the finite element results. As the wall thickness of the steel tube increased, the degree of local bulging gradually diminished, leading to a more uniform deformation distribution. In thick-walled specimens, no significant local instability was observed in the middle region of the column, with only minor local deformation occurring near the ends. Under the same wall thickness conditions, an increase in the strength of the core concrete resulted in a more localized post-peak deformation of the specimens, with the localization characteristics becoming more pronounced.

Figure 9 presents a comparison between the experimental and finite element load–displacement curves, demonstrating a strong correlation between the finite element predictions and the test results. During the initial loading stage, the stiffness of the simulated curves is slightly higher than that of the experimental curves, primarily due to the compaction of end gaps during testing. Once the peak load is surpassed, the finite element model effectively replicates the differences in response under varying parameters. Thin-walled specimens exhibit a more pronounced degradation after the peak load, while the post-peak curves become flatter as the wall thickness of the steel tube increases. Under the same wall thickness conditions, an increase in core concrete strength results in a significant enhancement of peak load capacity, but also leads to a more pronounced post-peak decline.

Table 6 compares the experimental ultimate bearing capacities with the finite element predictions for all specimens. The average value, standard deviation, and coefficient of variation of the *N_u_*/*N_m_* ratio are 1.02, 0.035, and 3.43%, respectively, with all predicted results falling within the ±10% error range. The close agreement in ultimate bearing capacity indicates that the finite element model incorporating the proposed confined DFCC constitutive relationship can reasonably reproduce the peak-load response of DFCC-filled steel tube stub columns.

Figure 9 compares the experimental and numerical load–displacement curves, and Table 7 summarizes the main response parameters. For each curve, the initial stiffness was represented by the secant stiffness at 40% of the corresponding peak load, *K*_0.4_ = 0.4*N_p_*/∆_0.4_. The displacement corresponding to *N_p_* was taken as the peak displacement. The residual capacity coefficient was defined as *η*_20_ *= N*_20_/*N_p_*, where *N*_20_ is the load at an axial displacement of 20 mm. To assess the overall curve agreement, the experimental and numerical curves were linearly interpolated at common displacement points from 0 to 20 mm. The normalized root mean square error was then calculated and normalized by the experimental peak load.

The experimental-to-numerical ratios of *η*_20_ ranged from 0.907 to 1.028, showing close agreement in residual capacity. The normalized root mean square errors ranged from 5.03% to 10.71%. For Ref-Q235-T3, D30F30-Q235-T8, and D20F30-Q235-T3, the peak-displacement ratios ranged from 0.966 to 1.094. The peak displacement showed greater variation for D30F30-Q235-T3 and D30F30-Q235-T5. The load changed gradually near the peak of these curves, making the displacement corresponding to the maximum load sensitive to small changes in load. For the thin-walled D30F30-Q235-T3 specimen, the localized deformation observed during the test also affected the measured axial displacement. Despite these differences, the normalized root mean square errors of D30F30-Q235-T3 and D30F30-Q235-T5 were 7.74% and 10.71%, and their residual-capacity ratios were 0.929 and 0.918, respectively. Their overall curves and post-peak responses therefore remained reasonably close to the test results.

The numerical curves showed higher initial stiffness because the idealized model assumed full contact from the beginning of loading, whereas the measured initial displacement included the compaction of small end gaps. This difference mainly affected the initial part of the curve and had little influence on the peak load or residual capacity.

Each entry gives the experimental value, numerical value, and experimental-to-numerical ratio. *K*_0.4_ is the secant stiffness at 40% of the corresponding peak load, and *η*_20_ is the residual capacity coefficient at an axial displacement of 20 mm.

Based on the comparisons of failure modes, load–displacement curves, and ultimate bearing capacities, the finite element model established with the proposed confined DFCC constitutive relationship can reasonably reproduce the axial compression behavior of DFCC-filled steel tube stub columns. The agreement in peak load and the overall response trend provides component-level support for the applicability of the proposed constitutive relationship within the investigated parameter range.

## 5. Conclusions

(1) The unconfined DFCC specimens displayed a pronounced post-peak softening behavior in their uniaxial compressive stress–strain curves. When the complete stress–strain curves were fitted using the Hu model, the correlation coefficients for all specimen groups exceeded 0.96, indicating that this model is suitable as the fundamental constitutive model.
(2) The confinement provided by steel tubes can enhance the load-carrying capacity, deformation capacity, and residual load-carrying capacity of DFCC. As the wall thickness of the steel tube increases from 3 mm to 5 mm and then to 8 mm, the confinement effect on the core concrete becomes progressively stronger. Consequently, the descending branch of the load–displacement curve following the peak load becomes flatter. This observation indicates that lateral confinement can effectively restrain the transverse deformation of DFCC and mitigate the risk of brittle failure.
(3) Based on the established confined constitutive models for lightweight aggregate concrete and considering the material characteristics of DFCC, the peak parameters and the descending branch were modified to develop a confined compressive constitutive model specifically applicable to DFCC. The proposed model reasonably describes the main characteristics of the ascending and descending branches within the investigated confinement range.
(4) The finite element results show reasonable agreement with the experimental results in terms of ultimate bearing capacity and overall load–displacement trend. The average ratio of experimental to simulated ultimate bearing capacity is 1.02, with a standard deviation of 0.035, indicating that the proposed constitutive relationship is applicable to the axial capacity analysis of DFCC-filled steel tube columns within the investigated parameter range.

Due to the limited number of specimens and the relatively narrow range of parameter combinations, further validation of the model is required when the main parameters differ substantially from the ranges investigated in this study. In addition, no friction-reducing measures were applied at the specimen ends in the present tests, which constitutes a limitation of this study and will be addressed in future work. Representative curves were adopted in this work, so the dispersion and repeatability of uniaxial-compression test results remain to be improved in future studies. The present study did not consider long-term or cyclic loading conditions. Therefore, future research should expand the experimental database to cover a wider range of DFCC mixture compositions, concrete strength grades, steel tube dimensions, steel grades, and confinement levels, and examine the model under long-term and cyclic loading conditions.

## Figures and Tables

**Figure 1:**
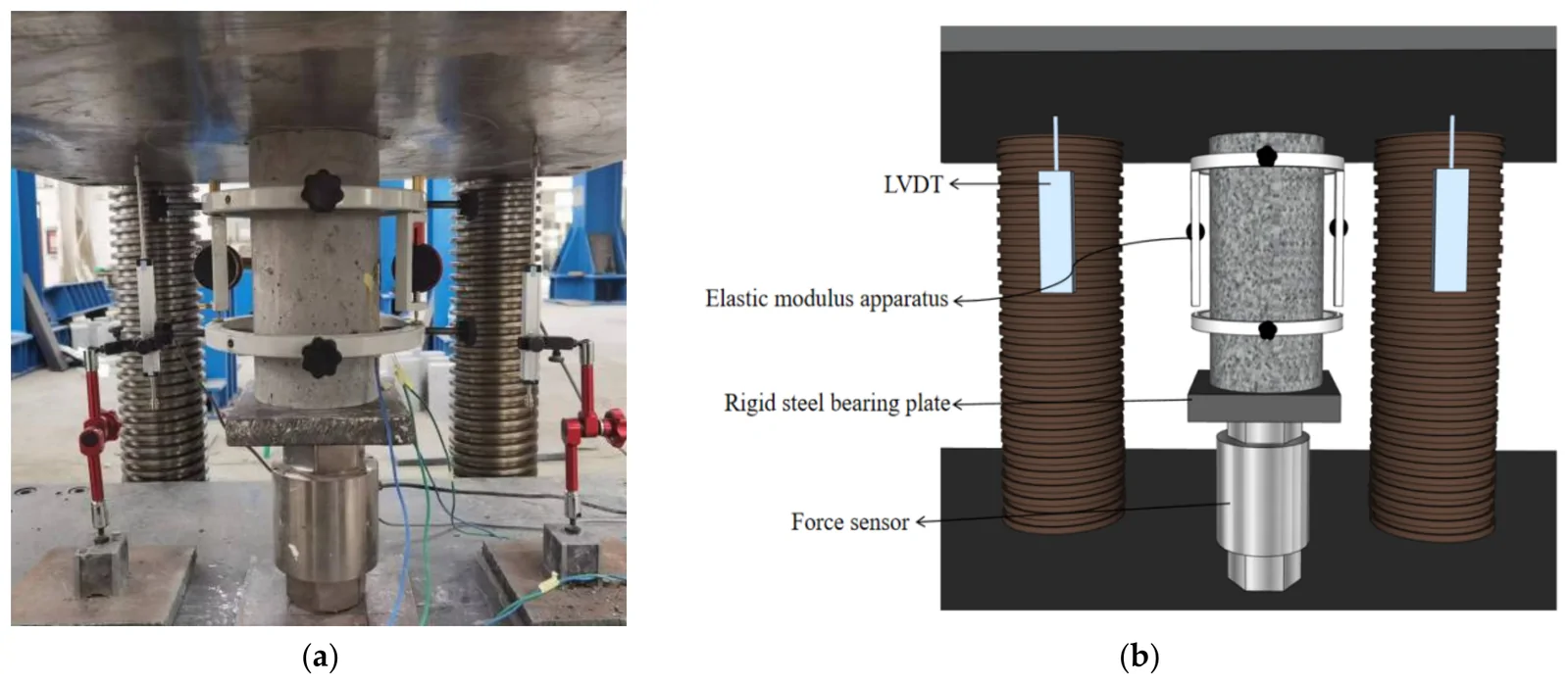
Diagram of the axial compressive strength test rig: (**a**) actual test setup; (**b**) schematic of the loading setup.

**Figure 2:**
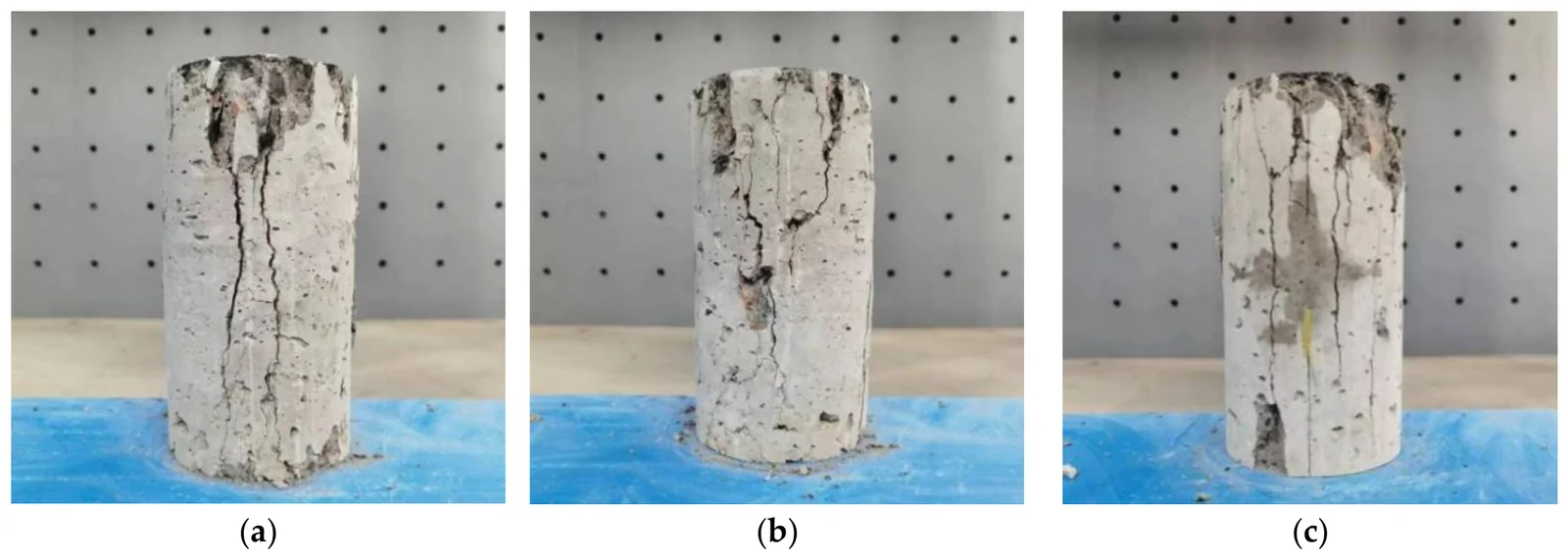
Failure patterns of DFCC specimens under uniaxial compression: (**a**) Ref-LC25; (**b**) D30F30-LC25; (**c**) D20F30-LC30.

**Figure 3:**
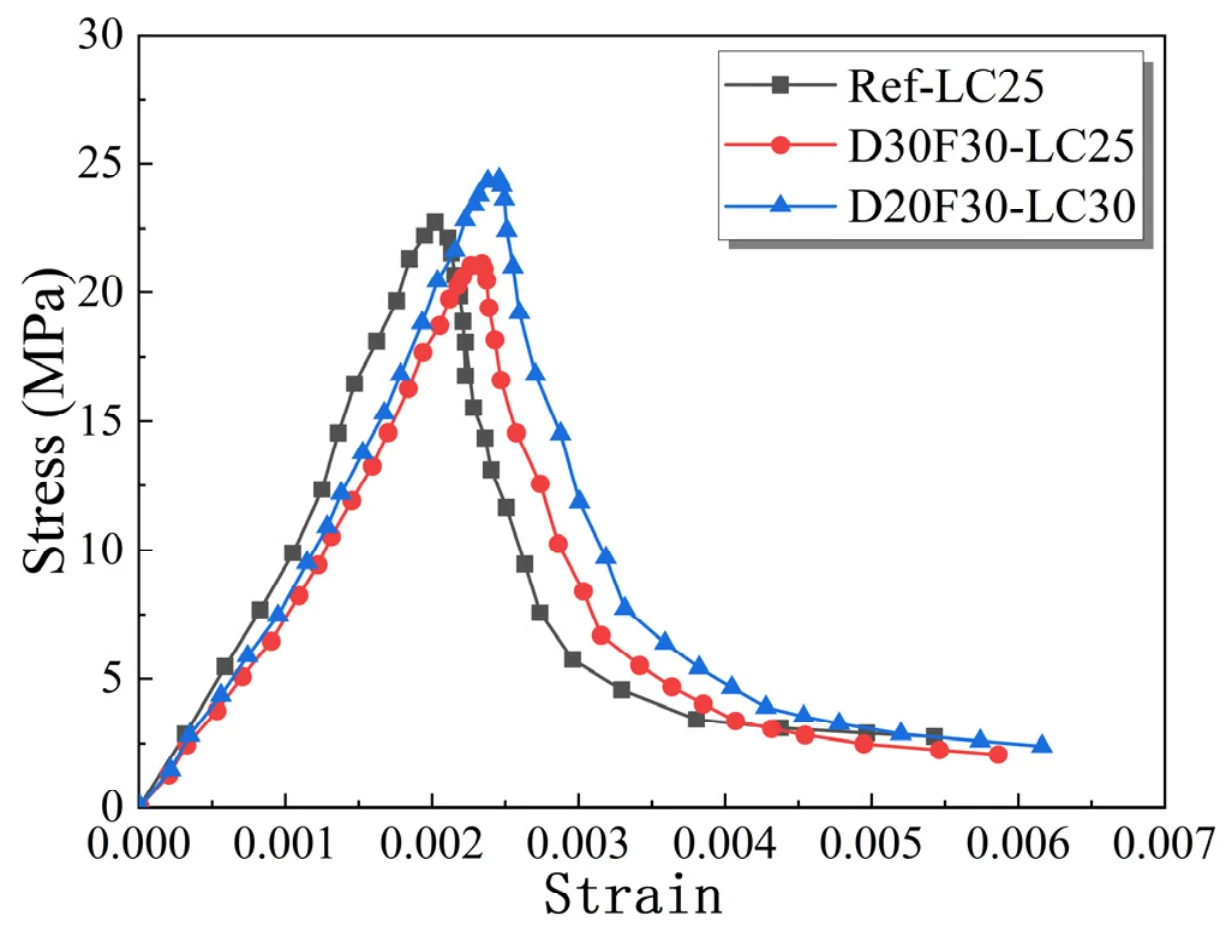
Stress–strain curves of DFCC under uniaxial compression.

**Figure 4:**
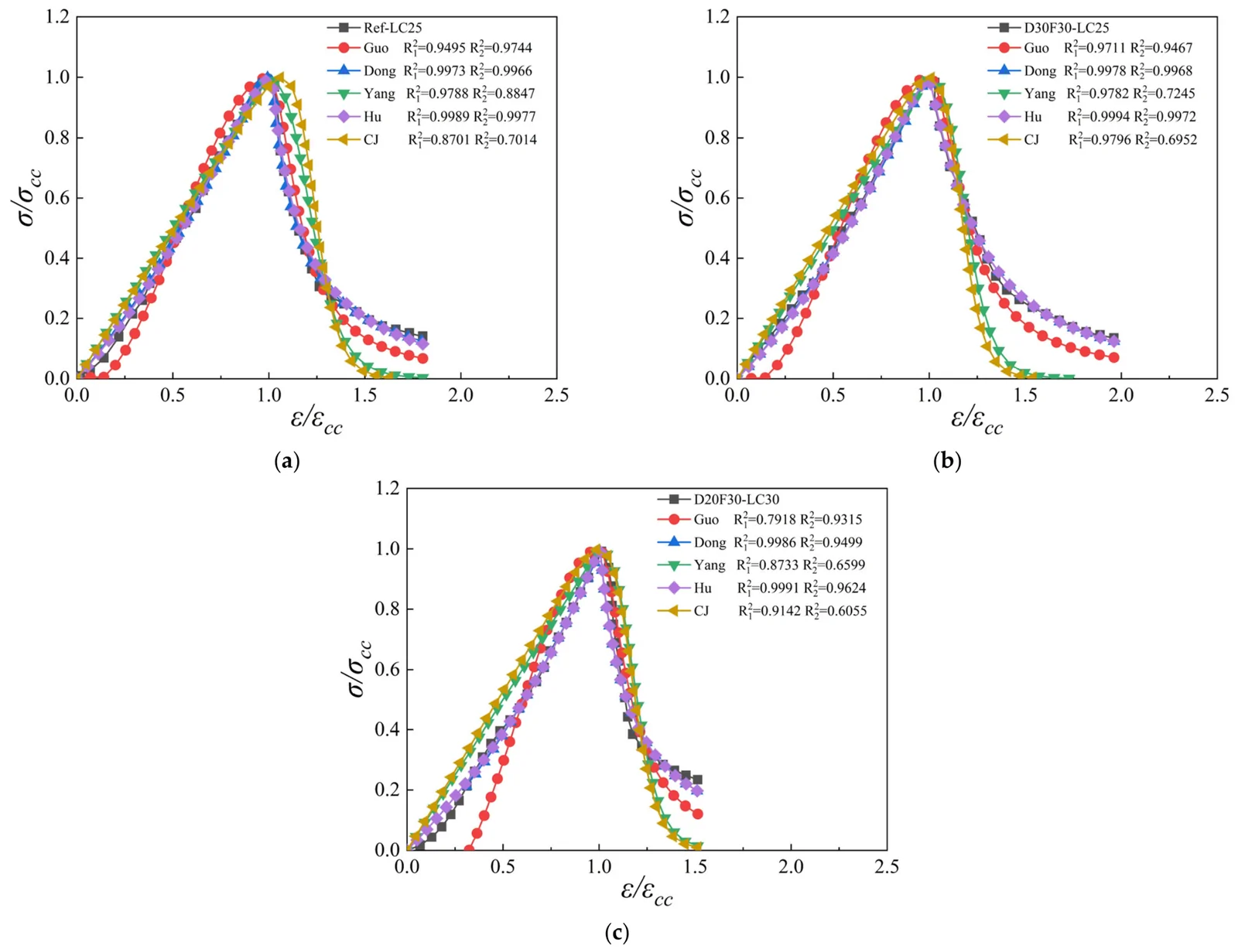
Comparison between experimental stress–strain curves and predictions from different constitutive models: (**a**) Ref-LC25; (**b**) D30F30-LC25; (**c**) D20F30-LC30 [31,32,33,34,35].

**Figure 5:**
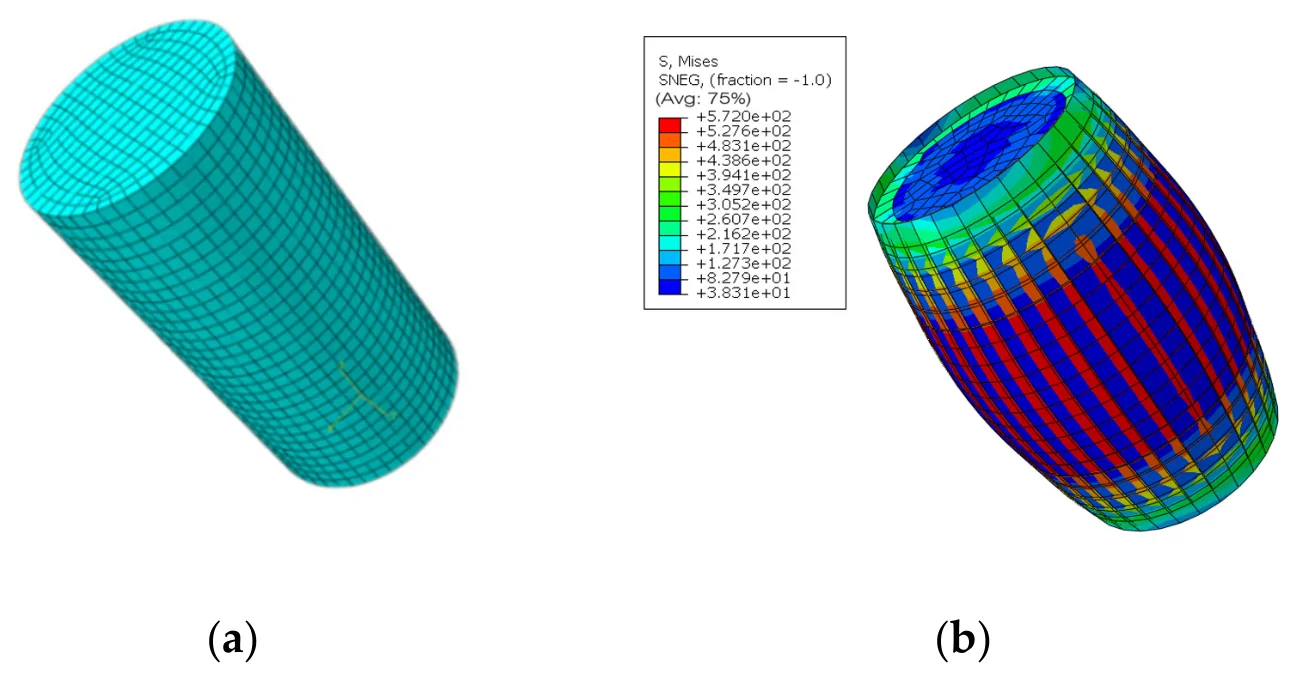
Schematic diagram of numerical simulation for concrete under steel tube confinement: (**a**) mesh division of core concrete; (**b**) core concrete under steel tube confinement.

**Figure 6:**
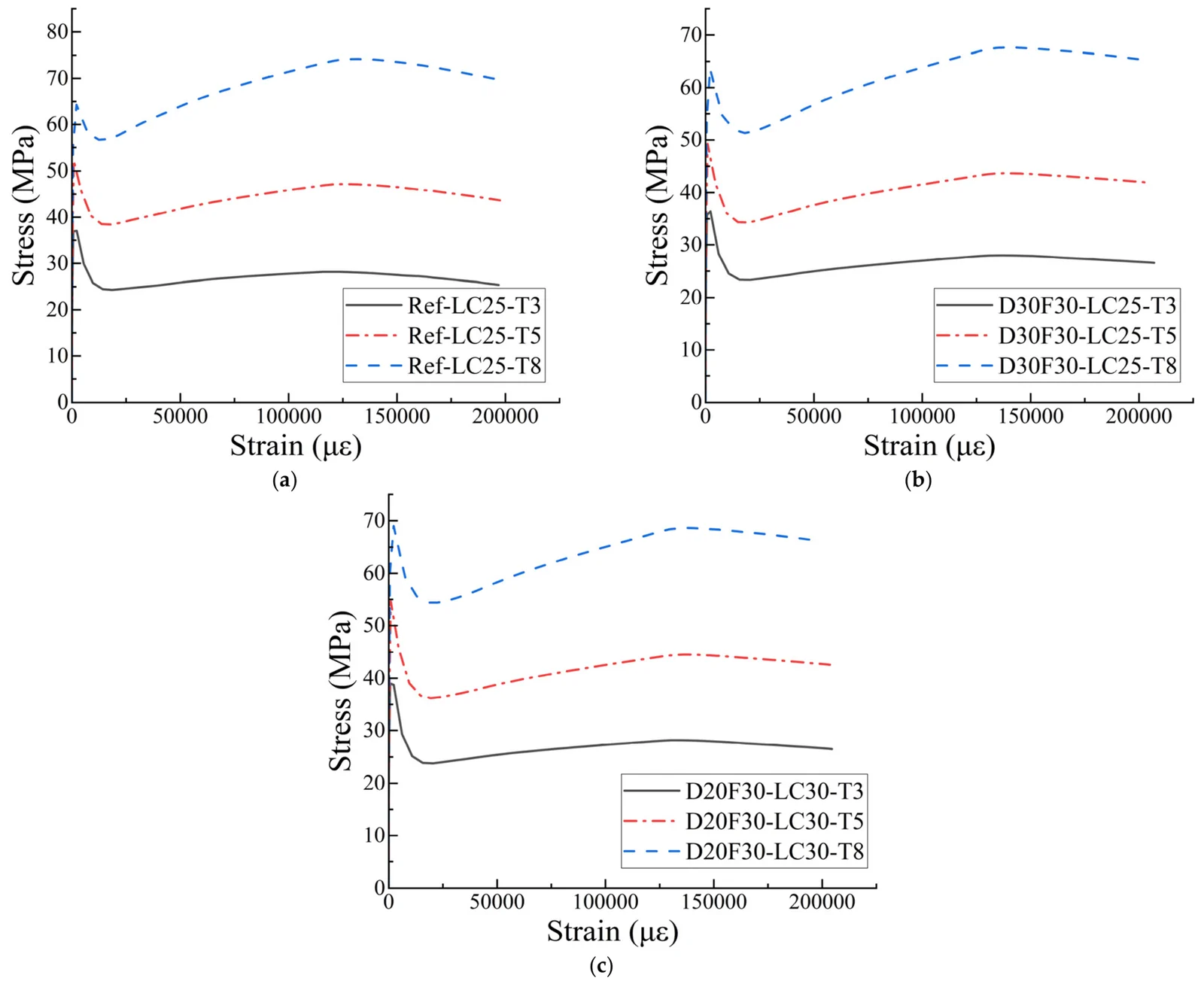
Simulated stress–strain curves of confined DFCC: (**a**) Ref-LC25; (**b**) D30F30-LC25; (**c**) D20F30-LC30.

**Figure 7:**
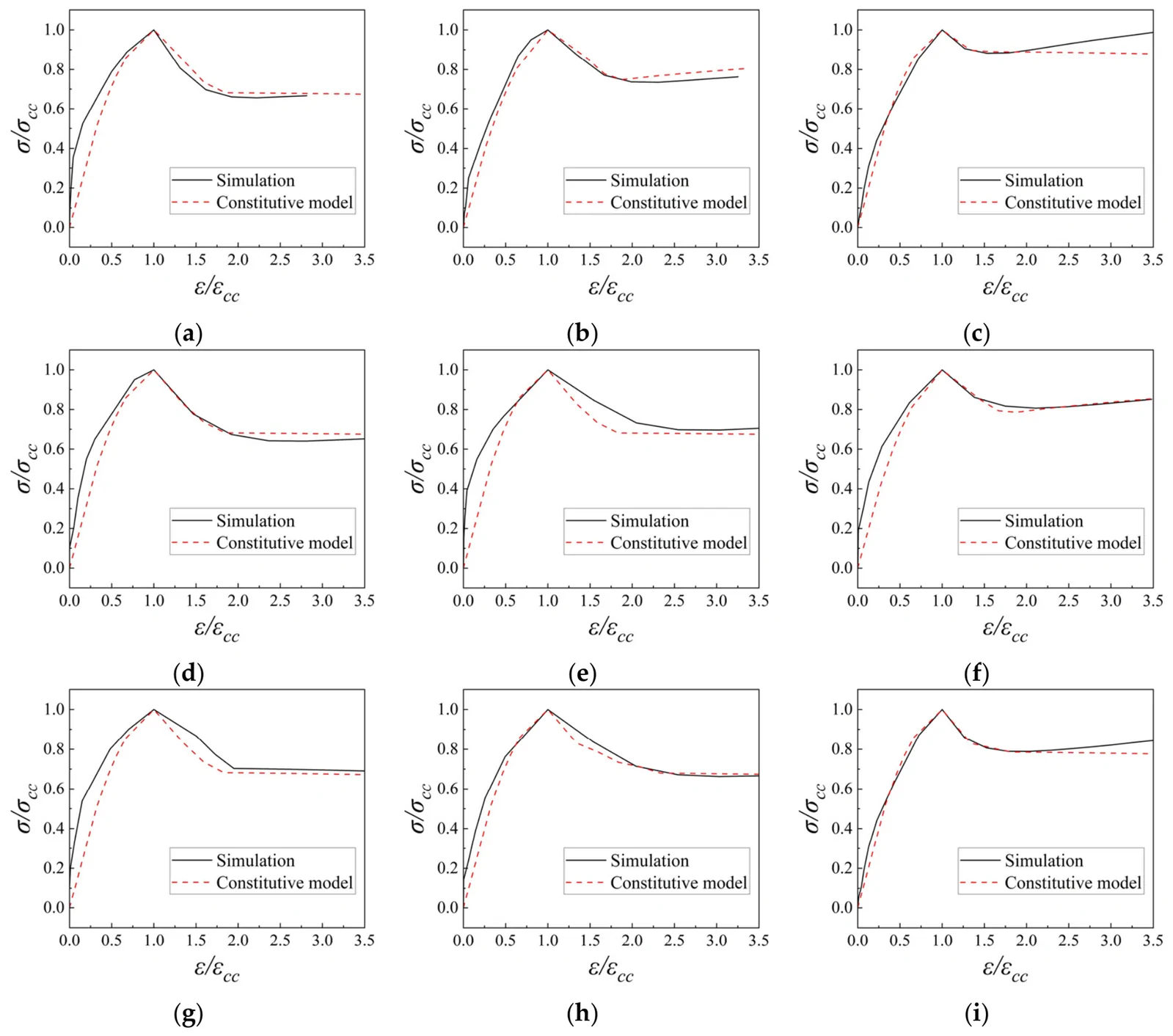
Comparison of the modified constitutive model with numerical simulation curves: (**a**) Ref-LC25-T3; (**b**) Ref-LC25-T5; (**c**) Ref-LC25-T8; (**d**) D30F30-LC25-T3; (**e**) D30F30-LC25-T5; (**f**) D30F30-LC25-T8; (**g**) D20F30-LC30-T3; (**h**) D20F30-LC30-T5; (**i**) D20F30-LC30-T8.

**Figure 8:**
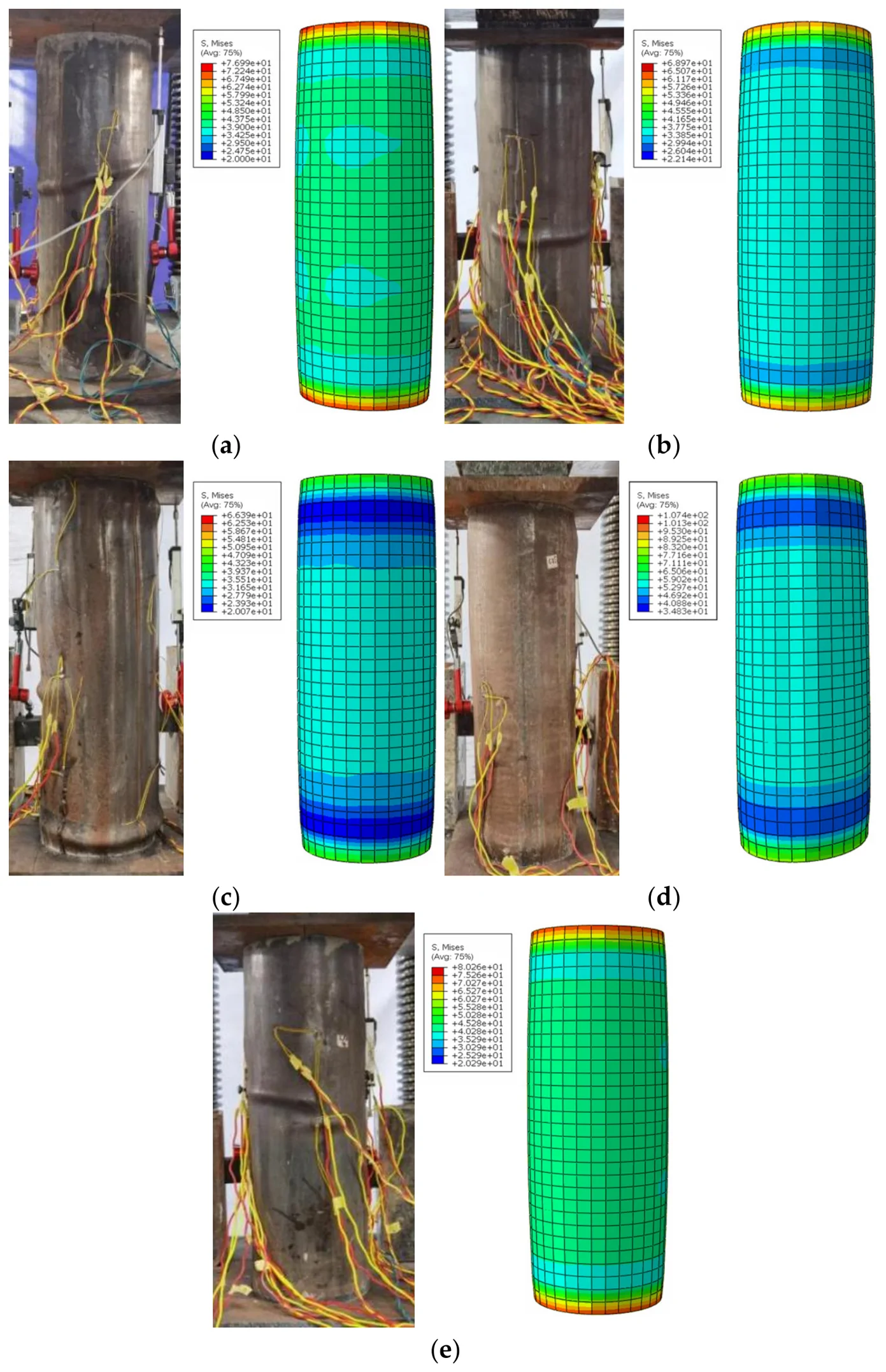
Comparison of test damage patterns and specimen simulation results: (**a**) Ref-Q235-T3; (**b**) D30F30-Q235-T3; (**c**) D30F30-Q235-T5; (**d**) D30F30-Q235-T8; (**e**) D20F30-Q235-T3.

**Figure 9:**
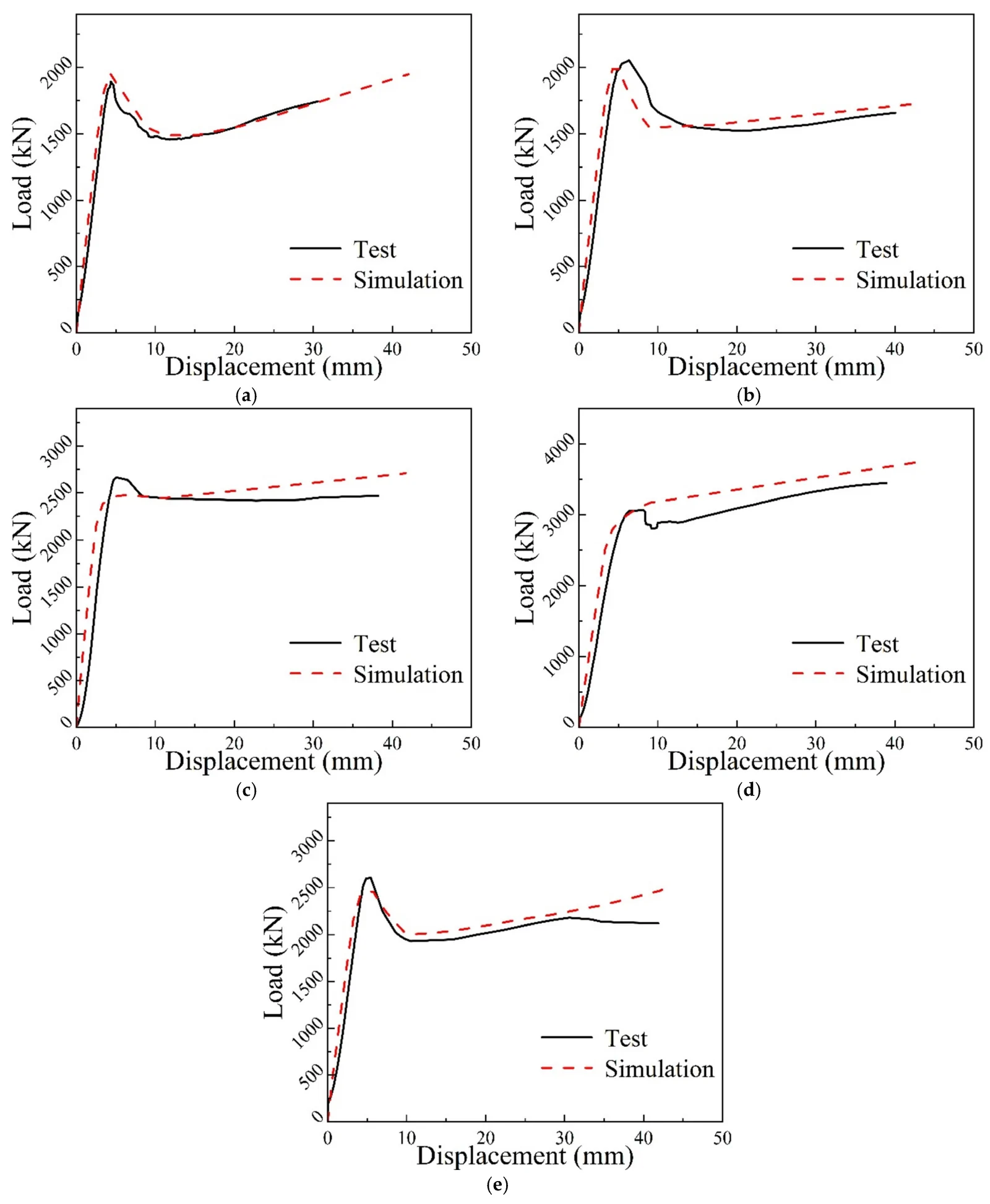
Comparison of experimental results with finite element load–displacement curves: (**a**) REF-Q235-T3; (**b**) D30F30-Q235-T3; (**c**) D30F30-Q235-T5; (**d**) D30F30-Q235-T8; (**e**) D20F30-Q235-T3.

**Table 1:** Physical properties of raw materials.

Material	Average Particle Size (mm)	Fineness Modulus	Apparent Density (kg/m^3^)	Bulk Density (kg/m^3^)
River sand	0.15–5.00	2.85	2475.00	1420.00
Desert sand	0.18	0.33	2620.00	1615.00
Fly-ash cenospheres	1.00–2.00	3.82	681.00	240.00
Shale ceramsite	5.00–20.00	-	1348.00	559.00

**Table 2:** Mix proportions of DFCC with different strength grades.

Specimen	Material Content (kg/m^3^)							
	Water	Cement	Fly Ash	Ceramsite	Desert Sand	River Sand	Fly-Ash Cenospheres	Polymer Emulsion
Ref-LC25	136.85	368.00	92.00	556.86	0.00	646.11	0.00	0.00
D30F30-LC25	112.94	345.92	92.00	556.86	220.29	258.44	51.87	45.99
D20F30-LC30	112.94	345.92	92.00	556.86	146.86	323.05	51.87	30.67

**Table 3:** Typical stress–strain constitutive model for concrete under compression.

Reference	Fitting Equation	Key Parameter Values
Guo model [31]	$y_{1}=\mathrm{ax}+(3-2a)x^{2}+(a-2)x^{3}$ $y_{2}=\frac{x}{b(x-1{)}^{2}+x}$	*a* is the ratio of the initial tangent modulus to the secant elastic modulus; *b* is determined according to the concrete strength grade and constraint condition.
Dong model [32]	$y_{1}=a_{1}x+{\;b}_{1}x^{2}$ $y_{2}=\frac{a_{2}x+{\;b}_{2}x^{2}}{1+\mathrm{cx}+dx^{2}}$	*a*_1_, *b*_1_, *a*_2_, *b*_2_, *c*, and *d* are all constants
Hu model [33]	$y=\frac{\mathrm{ax}+bx^{2}}{1+\mathrm{cx}+dx^{2}}$	*a*, *b*, *c*, *d* are all constants.
Yang model [34]	$y=\frac{(\beta_{1}+1)x}{x^{\beta_{1}+1}+\beta_{1}}$	$\beta_{1}=0.20\exp\left[0.73{\left(10/f_{c}\right)}^{0.67}{\left(w_{c}/2300\right)}^{1.17}\right]$ $\beta_{1}=0.41\exp\left[0.77{\left(10/f_{c}\right)}^{0.67}{\left(w_{c}/2300\right)}^{1.17}\right]$
Carreira-J model [35]	$y=\frac{\beta x}{\beta -1+x^{\beta }}$	$\beta =1/\left[1-\left(f_{c}^{\prime }/\varepsilon_{c}E_{c}\right)\right]$

**Table 4:** Fitted parameters of the Hu model for the complete stress–strain curves.

Group	a	b	c	d	R^2^
Ref-LC25	0.7177	−0.5794	−1.2160	0.3545	0.9646
D30F30-LC25	0.6617	−0.6041	−1.3275	0.3852	0.9734
D20F30-LC30	0.6716	−0.5321	−1.4843	0.3261	0.9794
Mean					0.9725
Standard deviation					0.0061

**Table 5:** Design parameters of DFCC-filled steel tube stub columns.

Specimen	D (mm)	T (mm)	L (mm)	Slenderness Ratio	Concrete Strength	*f*_c_ (MPa)	*f*_y_ (MPa)	θ	N_u_ (kN)
Ref-Q235-T3	219	3	650	2.96	LC25	22.73	271	0.77	1894.6
D30F30-Q235-T3	219	3	650	2.96	LC25	21.12	271	0.93	2055.8
D30F30-Q235-T5	219	5	650	2.96	LC25	21.12	345	1.18	2589.3
D30F30-Q235-T8	219	8	650	2.96	LC25	21.12	420	1.43	3054.7
D20F30-Q235-T3	219	3	650	2.96	LC30	24.41	270	0.77	2605.3

**Table 6:** Comparison of ultimate load capacities between test and finite element simulation.

Specimen	*N*_u_ (kN)	*N*_m_ (kN)	*N*_u_/*N*_m_
Ref-Q235-T3	1894.69	1950	0.97
D30F30-Q235-T3	2055.88	1990	1.03
D30F30-Q235-T5	2589.31	2474	1.04
D30F30-Q235-T8	3054.71	3034	1.01
D20F30-Q235-T3	2605.32	2457	1.06
Average			1.02
Standard Deviation			0.035
Coefficient of Variation			3.43%

**Table 7:** Quantitative comparison of experimental and numerical load–displacement responses.

Specimen	*K*_0.4_, Test/FE (Ratio) (kN/mm)	Peak Displacement, Test/FE (Ratio) (mm)	*η*_20_, Test/FE (Ratio)	NRMSE (%)
Ref-Q235-T3	438.7/570.0 (0.770)	4.37/4.34 (1.006)	0.817/0.795 (1.028)	5.03
D30F30-Q235-T3	407.5/564.5 (0.722)	6.33/4.26 (1.486)	0.742/0.798 (0.929)	7.74
D30F30-Q235-T5	497.5/948.5 (0.525)	4.54/5.99 (0.757)	0.937/1.020 (0.918)	10.71
D30F30-Q235-T8	535.1/793.0 (0.675)	6.34/6.56 (0.966)	1.013/1.106 (0.915)	10.54
D20F30-Q235-T3	507.3/712.9 (0.712)	5.45/4.98 (1.094)	0.773/0.853 (0.907)	5.79

## Data Availability

The original contributions presented in this study are included in the article. Further inquiries can be directed to the corresponding authors.
